# Evaluation of atmospheric correction algorithms for salt lake water assessment: Accuracy, band-specific effects, and sensor consistency

**DOI:** 10.1371/journal.pone.0315837

**Published:** 2024-12-23

**Authors:** Changjiang Liu, Fei Zhang, Chi-Yung Jim, Saheed Adeyinka Oke, Elhadi Adam

**Affiliations:** 1 Xinjiang Laboratory of Lake Environment and Resources in Arid Zone, Urumqi, China; 2 College of Geographic Science and Tourism, Xinjiang Normal University, Urumqi, China; 3 College of Geography and Environmental Sciences, Zhejiang Normal University, Jinhua, China; 4 Department of Social Sciences, Education University of Hong Kong, Tai Po, Hong Kong, China; 5 Civil Engineering Department, Central University of Technology, Bloemfontein, South Africa; 6 School of Geography, Archaeology and Environmental Studies, University of the Witwatersrand, Johannesburg, South Africa; Atlantic Technological University, IRELAND

## Abstract

Atmospheric correction plays an important role in satellite monitoring of lake water quality. However, different atmospheric correction algorithms yield significantly different accuracy for inland lake waters beset by shallowness and turbidity. Finding a suitable algorithm for a specific lake is critical for quantitative satellite water-environmental monitoring. This study used Landsat 8 and Sentinel 2 L1 level data of Ebinur Lake in arid northwest China on May 19, 2021. Atmospheric corrections were performed using FLAASH, QUAC, 6S, Acolite-DSF and Acolite-EXP algorithms. The Sentinel 2 reflectance product verified the consistency of the algorithms. Quasi-simultaneously measured hyperspectral data determined the algorithm applicable to Ebinur Lake waters. The results indicate that the Acolite-DSF algorithm has good consistency and high accuracy in the atmospheric correction of Landsat 8 and Sentinel 2 images. Extracting the atmospheric correction of Landsat 8 images found relative error at 0.3 in the Blue, Green, and Red bands and 0.5 in the NIR band. For comparison, the relative errors of Sentinel 2 in all bands are 0.3. Therefore, these four bands of Landsat 8 and Sentinel 2 data are recommended for temporal monitoring of water-environmental parameters in Ebinur Lake. Besides identifying the suitable atmospheric correction algorithm for Ebinur Lake, this study analyzed the atmospheric correction errors of common wavebands for remote sensing monitoring of water bodies, especially applicable for inland salt lakes of arid regions.

## 1. Introduction

Monitoring the environmental parameters of lake waters, reflecting their quality, has notable practical significance for utilization, management, conservation and restoration. Remote sensing offers an effective synoptic tool for such monitoring work. However, solar radiation is absorbed and scattered by aerosols, air molecules, pure water, and water components before satellite sensors receive it. Thus, the water signals received by the sensors are often submerged or veiled by atmospheric signals [1]. Therefore, it is essential to effectively remove the atmospheric signals by correction measures to extract the radiation signals of water components [2].

The most widely used atmospheric corrections are based on the theory of atmospheric radiative transfer [3]. Traditional atmospheric correction algorithms for water bodies include the near-infrared (NIR) and its improved versions for specialized application to clean ocean water bodies. Such algorithms assume zero radiation away from water in the NIR band to obtain the reflectance in each band [4–6]. Subsequently, shortwave infrared atmospheric correction algorithms were developed for turbid inland and nearshore waters [7, 8]. These algorithms are integrated into NASA’s water color remote sensing processing software SeaDAS (SeaWiFS Data Analysis System). It can be used for atmospheric correction of water bodies for ocean water color sensors such as MODIS, GOCI, VIIRS, and SeaWiFS, and terrestrial sensors such as Sentinel 2 and Landsat series [4, 7]. In addition, similar principles of atmospheric correction algorithms have been integrated into Acolite software [8, 9], applicable to atmospheric correction of Sentinel 2 and Landsat series satellites for inland lakes. This method has received extensive attention [10–13]. Especially in turbid waters, the Acolite atmospheric correction algorithm can demonstrate good performance [14]. Wu et al. (2023) proved its stability in time sequence in the turbid waters of the the Pearl River Estuary [15].

Research has shown that various atmospheric correction methods have different applicability scenarios[16]. For example, Acolite shows excellent performance in the red band [17], while C2RCC shows inconsistencies in the blue and green bands [18]. FLAASH excels in handling OLCI data, while iCOR shows overcorrection at lower resolutions [19]. There are again atmospheric correction algorithms that are known for providing a large range of reflectance product data, such as L2gen [20]. Inland lakes, usually with large shallow water areas, have relatively complex optical conditions and variable aerosol compositions. The generation of off-water radiation signals jointly contributed by water column components and underwater sediments [3] has limited the efficacy of atmospheric correction algorithms [21]. Relevant studies have yielded promising results on atmospheric correction for lake waters [22]. Atmospheric correction algorithms are based on three main methods: (1) graphic and image processing; (2) inversion of atmospheric parameters from image information; and (3) synchronized atmospheric corrector [23].

The graphical and image processing method does not focus on the physical mechanism. Instead, it adopts the "clear distorted air" or "defog" algorithm for atmospheric correction [24], regarded as a relative atmospheric correction approach. The inversion of atmospheric parameters method is based on image information. It does not need to obtain atmospheric parameters derived from actual measurements. Instead, it extracts atmospheric parameters from the image itself. With a very high degree of automation, it has been widely applied to the Gaofen, ZY and HJ satellites launched by China [25].

The synchronized atmospheric corrector approach considers the characteristics of atmospheric transients. It employs on-board small, specialized atmospheric sounders to acquire atmospheric parameters synchronously and capture reflectivity information in specified bands. It has gradually become an important atmospheric correction tool [26]. Other methods have been developed for atmospheric correction of lake water bodies, such as the neural network [27], principal component analysis [28], and integrated switching [29]. Incomplete atmospheric corrections (e.g., corrections for Rayleigh scattering only) have been employed [30].

The applicability of atmospheric correction algorithms depends on their sensitivity to atmospheric components and tolerance to noise. There are also differences in their performance when dealing with different geographical regions and atmospheric conditions [31]. In remote sensing monitoring of the water environment, the applicability of atmospheric correction algorithms varies considerably by region [32]. Failure to optimize the atmospheric correction algorithms for a specific target will bring uncertainty to remote-sensing monitoring of water-environmental parameters.

The Ebinur Lake in China’s arid northwest region was chosen as the study area. It is a salt lake surrounded by extensive saline soil cover [33]. Wind-suspended atmospheric aerosols are significantly different from other regions due to the inclusion of salt dust [22]. The effect of using the same atmospheric correction algorithm (such as FLAASH) for different remote sensing data in the Ebinur Lake area is not ideal [29]. To obtain more accurate and continuous water environment parameters, it is still necessary to find suitable atmospheric correction methods [24]. This study aimed at three objectives: (1) Comparing the accuracy of selected atmospheric correction algorithms in assessing the waters of the salt lake; (2) Analyzing the effect of atmospheric correction in different bands of the sensors. (3) Examining the consistency of each atmospheric correction algorithm applied to different sensors. The research results are expected to provide reference for remote-sensing monitoring of water environment parameters in salt lakes in arid areas.

## 2. Materials and methods

### 2.1 The study area

**Fig 1:**
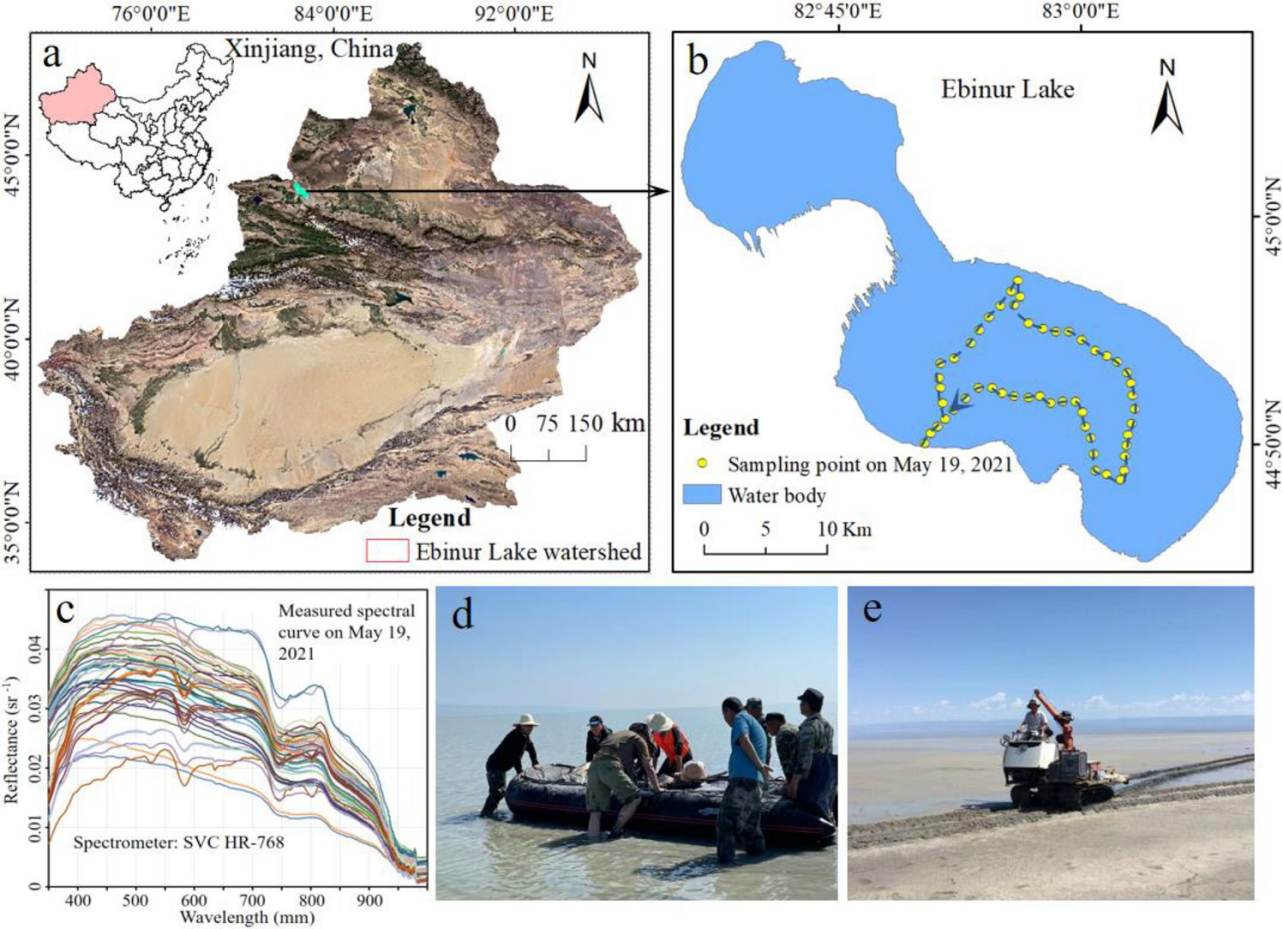
Study area and sampling (a: The location of Ebinur Lake in northwest China; b: Distribution of 52 sampling points; c: Water spectral curve; d: The research team entering the lake with field measuring equipment installed on a boat; e: The crawler carrying measuring equipment. The acquisition address of the remote sensing image in Fig 1a is http://viewer.nationalmap.gov/viewer/).

Ebinur Lake (N44°20′−45°45′, E82°35′−83°10′) is a typical salt lake in northwest China, with an altitude of about 189 m (Fig 1). The annual average temperature in the Ebinur Lake area is 7.8°C. It is one of the richest lakes in China in terms of halophyte resources [34]. The water is shallow (average depth of about 120 cm) and turbid (average transparency of only 17.51 cm in the summer and fall of 2009−2018) [35]. Its water has a density of about 1.08 g/cm^3^, pH of about 7.98, and salinity of about 169.3 g/L. The water quality is the most eutrophic of important lakes in China (Ministry of Ecology and Environment of the People’s Republic of China, https://www.mee.gov.cn/).

The region’s climate is dry, with an average annual precipitation of less than 10 mm from late spring to mid-fall, often accompanied by strong windy weather [35]. The intermittently exposed lake bed of Ebinur Lake is extensive, and the composition of the dry lake bed material is mainly lake-phase loose salt-rich fine-grained sediments, which are highly susceptible to salinization. Under wind action, salt dust storms are frequently triggered, imposing a serious obstacle to the sustainable development of oasis agriculture in arid and semi-arid areas.

### 2.2 Water sampling design and spectral measurement

The field data collection of the manuscript has received full support from the Bolata Mongol Autonomous Prefecture Forestry and Grassland Bureau. The sampling routes were determined according to lake water conditions and the distribution of sampling points shown in Fig 1. The sampling routse was determined based on the draft depth of the boat and the depth of the lake. Sampling was conducted on May 19, 2021, and the locations of the 52 sampling sites are shown in Fig 1. Water samples were collected following the Technical Specification Requirements for Surface Water and Wastewater Monitoring [36]. A sampler collected 500 mL water samples about 0.2 m below the surface. The water samples were placed in polytetrafluoroethylene (PTFE) plastic bottles and temporarily stored in a benzene sheet insulated box filled with ice cubes. After each boat trip, the samples were promptly transported to the laboratory within one day for parameter testing.

Field measurements were carried out after the boat was stabilized, and the GPS coordinates of the sampling points were recorded. Ten points of hyperspectral data were obtained by the in-situ measurement method. An ASD fieldspec3 ground object spectrometer (Analytical Spectral Devices, Boulder, CO, USA) was used to obtain hyperspectral data. The spectrometer has a wavelength range of 350−2500 nm and provides 768 spectral bands, with a spectral resolution of 1 nm in the 480−980 nm range. The hyperspectral probe was placed approximately 1.2 m from the water surface [37–39]. At each sampling point, at least 10 measurements were made, and the average of the normal spectral curves was taken as the measured hyperspectral data. The spectral data were checked line by line during the measurement, and the normal data were numbered and organized. The ViewSpecPro (version 6.0) software was used to normalize the data and remove the edge bands. The normalized hyperspectral data still have "burrs" because of the difference in energy response in each band. This study employed the Savitzky-Golay filter smoothing method to realize the denoising and smoothing of the spectral curve [31]. Finally, the remotely sensed reflectance (R_rs_) is obtained by:

$$R_{rs}=\left[L_{sw}(\lambda )-\gamma L_{sk}(\lambda )\right]\rho_{p}(\lambda )/\pi L_{p}(\lambda )$$ (1)

where *L*_*sk*_(*λ*), *L*_*sw*_(*λ*) and *L*_*p*_(*λ*) are the irradiance values of the sky, the measured water surface, and the standard whiteboard, respectively; γ is the air-water interface reflectance, which takes the value of 0.022 for a calm water surface; and ρ_*p*_(*λ*) is the standard whiteboard reflectance.

### 2.3 Satellite image acquisition

To fully evaluate the applicability of atmospheric correction methods to the waters of Ebinur Lake, this study performed multiple atmospheric corrections on Landsat 8 and Sentinel 2 images. Their images with high spatial resolution have been widely used in remote-sensing monitoring of water-environmental parameters of inland lakes [11, 32]. Landsat 8 images are mainly obtained from the Geospatial Data Cloud Platform (http://www.gscloud.cn/search), and the Sentinel 2 images and their reflectance products are mainly obtained from EROS (https://www.usgs.gov/centers/eros). Although both Landsat 8 and Sentinel 2 transited Ebinur Lake on May 19, 2021, the transit moments of the two sensors were different.

Based on the experience of previous studies [28, 29], the commonly used bands for remote sensing monitoring of water-environmental parameters are Blue, Green, Red, NIR, SWIR1, and SWIR2. Therefore, this study focused on the atmospheric correction effect of these six bands. The central wavelengths of the six bands in Landsat 8 (Sentinel 2) are 483 (492), 561 (560), 655 (665), 865 (833), 1610 (1614), and 2200 (2202) nm, respectively.

### 2.4 Atmospheric correction methods

Different atmospheric correction methods can lead to inconsistencies in the accuracy of water-environmental parameters, compromising the reliability of remote sensing monitoring. This study proposed four atmospheric corrections, namely FLAASH, QUAC, 6S and Acolite (DSF and EXP), for application to Ebinur Lake. Sentinel 2 reflectance products were used to check the consistency of these atmospheric correction algorithms for different sensors, and the measured hyperspectral data were used to validate the absolute accuracy. All atmospheric correction methods can be found in publicly available code on GitHub.

#### 2.4.1 FLAASH atmospheric correction

Since integrating the FLAASH (Fast Line-of-sight Atmosphere Analysis of Spectral Hypercubes) algorithm into ENVI (The Environment for Visualizing Images), it has become one of the most used atmospheric correction approaches. The algorithm uses the MODTRAN4^+^ (MODerate spectral resolution atmospheric TRANsmittance) model to calculate the empirical values of atmospheric parameters input to the radiative transfer equations. It uses the discrete longitudinal coordinate method (DISORT) to solve the equations to obtain the surface reflectance. It performs pixel-by-pixel correction of the radiance bias caused by atmospheric molecules such as CO_2_, O_2_, O_3_ or aerosols in the atmosphere [40]. It can effectively eliminate most atmospheric interferences to obtain accurate surface reflectance data and is suitable for atmospheric correction of remote sensing images in the 0.4~3 μm range [41–43]. For Landsat 8, the FLAASH was performed with a sensor height of 705 km, a ground elevation of 0.2 km, an atmospheric mode of mid-latitude summer, an aerosol model of Rural, and the K-T method of aerosol inversion. For Sentinel 2, the sensor height was 786 km, and the other parameters were the same as above.

#### 2.4.2 QUAC atmospheric correction

The QUAC (Quick Atmospheric Correction) atmospheric correction algorithm differs from FLAASH because it does not need to input the sensor and atmospheric parameters manually. Instead, it reads the atmospheric compensation parameters directly from the target image (target pixel spectrum). It can obtain accurate surface reflectance data without atmospheric observation records. QUAC is an empirical algorithm based on the dark target detection method. It obtains the experience to complete the fast atmospheric correction by automatically collecting the spectral information of different features on the remotely sensed image [44].

#### 2.4.3 6S atmospheric correction

The 6S (Second Simulation of the Satellite Signal in the Solar Spectrum) radiative transfer model is based on an improved version of the 5S model. It is suitable for atmospheric corrections in the 250 to 4000 nm range. It is calculated using the successive and approximate scattering algorithms synthesizing the subsurface conditions, elevation and other factors in the study area when performing aerosol scattering and atmospheric molecular scattering inversions. This study implemented the atmospheric correction for the waters of Ebinur Lake according to Vermote’s method [45]. The atmospheric model was mid-latitude summer, the aerosol model was continental, the optical thickness of 550 nm was used for aerosol concentration, and the sensor altitude code was set to -1000.

#### 2.4.4 Acolite atmospheric correction

Two kinds of atmospheric correction algorithms are built into Acolite (Atmospheric Correction for Operational Land Imager ‘lite’), namely the dark spectrum fitting (DSF) method [46–48] and the exponential extrapolation (EXP) method [7, 8].

EXP embeds an atmospheric correction method based on the atmospheric radiative transfer model in three band combinations: Red/NIR, NIR/SWIR1, and SWIR. This study adopted the NIR/SWIR1 band combination, validated in the time series monitoring of water color parameters in lakes in the middle and lower reaches of the Yangtze River [49]. Based on NIR/SWIR1, the exponential extrapolation method can effectively weaken the effect of θ not being constant due to the higher absorption of the water body in the SWIR1 band [50]. The assumptions of the NIR/SWIR1 index-based extrapolation method are similar to the classical atmospheric correction method [3], and the aerosol reflectance in other bands is extrapolated on this basis [7].

DSF was initially embedded into Acolite for atmospheric correction of meter-scale spatially resolved remote sensing data [46]. The DSF atmospheric correction algorithm calculates the atmospheric range radiance reflectance (*ρ*_*path*_) based on the assumption of zero reflectance for a sensor band within the field or sub-field of view without predefining the dark bands. The histogram of top-of-atmosphere reflectance (*ρ*_*toa*_) is computed, and then ordinary least squares regression is applied to the first 1,000 image elements with low reflectance values. Finally, its intercept represents the best reflectance estimate for the darkest target in the band to be corrected (*ρ*_*dark*_). A pre-generated look-up table is used to compare *ρ*_*path*_ and *ρ*_*dark*_, and the continental or oceanic aerosol type is selected based on the principle that the RMSD (root-mean-square deviation) between *ρ*_*dark*_ and *ρ*_*path*_ is lowest for the two closest bands. The mask wavelength for EXP and DSF was 1600 nm, the mask threshold was set to 0.0215 *sr* ^-1^, and the flare mask threshold was 0.05 *sr* ^-1^.

### 2.5 Accuracy evaluation

This study examined the absolute accuracy of various atmospheric correction algorithms using measured hyperspectral data to explore the atmospheric correction methods suitable for the waters of Ebinur Lake [17]. The evaluation indexes of atmospheric correction accuracy of different sensors include the coefficient of determination (*R*^*2*^), mean absolute percentage error (*MAPE*, the smaller its value, the higher the accuracy), and *Slope* (the closer the value to 1, the higher the accuracy). Their equations are:

$$R^{2}=\frac{{\left(\sum \left(X_{i}-\bar{X}\right)\left(Y_{i}-\bar{Y}\right)\right)}^{2}}{\sum {\left(X_{i}-\bar{X}\right)}^{2}\sum {\left(Y_{i}-\bar{Y}\right)}^{2}}$$ (2)

$$MAPE=\frac{1}{n}\sum_{i=1}^{n}\left|\frac{Y_{i}-X_{i}}{X_{i}}\right|\times 100\%$$ (3)

$$Slope=\frac{\left(Y_{2}-Y_{1}\right)}{\left(X_{2}-X_{1}\right)}$$ (4)

where Y represents the atmospherically corrected remotely sensed reflectance value, X represents the measured hyperspectral value, and $\bar{X}$ and $\bar{Y}$ represent the mean values of the measured hyperspectral and atmospherically corrected remotely sensed reflectance, respectively.

## 3. Results

### 3.1 Quantitative evaluation of atmospheric calibration results

**Fig 2:**
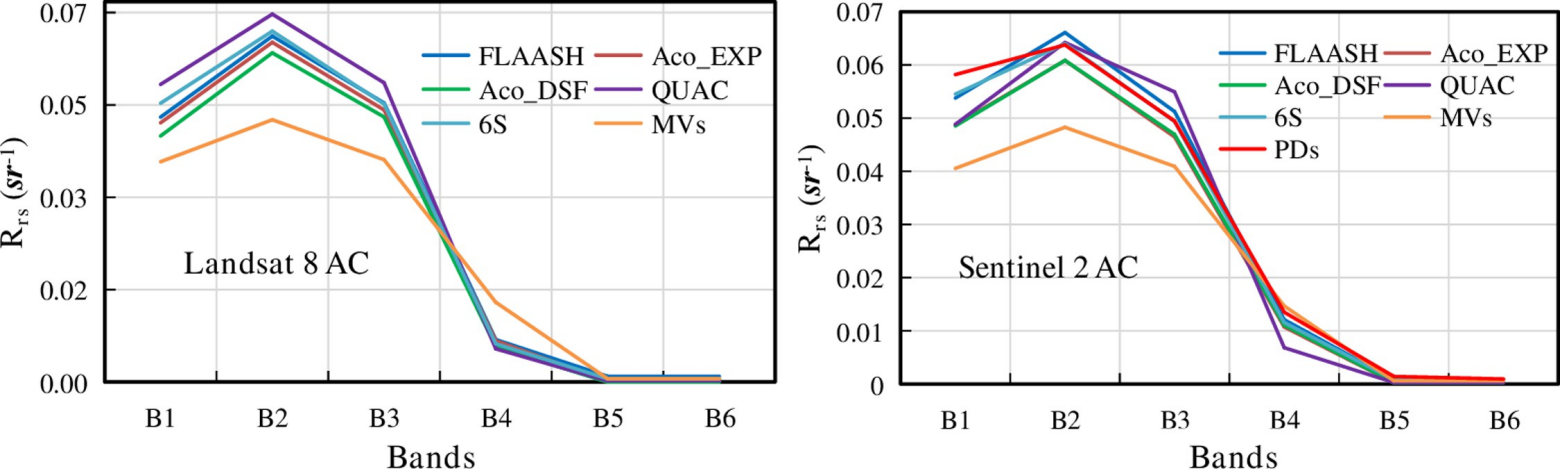
Comparing the curves between atmospheric correction and measured reflectance values for Landsat 8 and Sentinel 2 (AC: Atmospheric Correction; MVs: Measured Value; Aco: Acolite; PDs: Production Data).

Hyperspectral reflectance values were obtained on May 19, 2021, at 52 sampling points. The center wavelengths of the Blue, Green, Red, NIR, SWIR 1, and SWIR 2 bands of the Landsat 8, and Sentinel 2 are labeled as B1, B2, B3, B4, B5, and B6, respectively. The average of the measured hyperspectra of the 52 points was taken as the reflectance value at the corresponding center wavelength. Then, the reflectance at the center wavelength of the six bands was extracted for each image after atmospheric correction. The reflectance values of Landsat 8 and Sentinel 2 after atmospheric correction were plotted against the measured values (Fig 2).

Fig 2 shows that the reflectance values of the measured hyperspectral curves in the first three bands are generally lower than those of Landsat 8 and Sentinel 2 after atmospheric correction. The opposite trend is observed in the last three bands. Hyperspectral reflectance was tested indoors, while reflectance during satellite transit was produced in a natural environment. At the first three wavelengths, the satellite transit is affected by the bottom effect and banked diffuse reflection, resulting in higher reflectance values than the measured values. In the last three bands, the bottom effect and shoreline diffuse reflection were greatly weakened, resulting in lower remotely sensed reflectance than measured values. The trends of the curves of different atmospheric correction algorithms for Landsat 8 are the same. Among them, Acolite-DSF and Acolite-EXP are the closest to the measured values, the curves overlap, and it is difficult to distinguish their accuracy from that of Fig 2. The atmospheric correction curves of Sentinel 2 are similar, but the difference between their B1 band and reflectance product data is more obvious. The Acolite-DSF and Acolite-EXP corrections are also closest to the measured values, but it is still difficult to distinguish the accuracy differences between the two algorithms in Fig 2.

**Fig 3:**
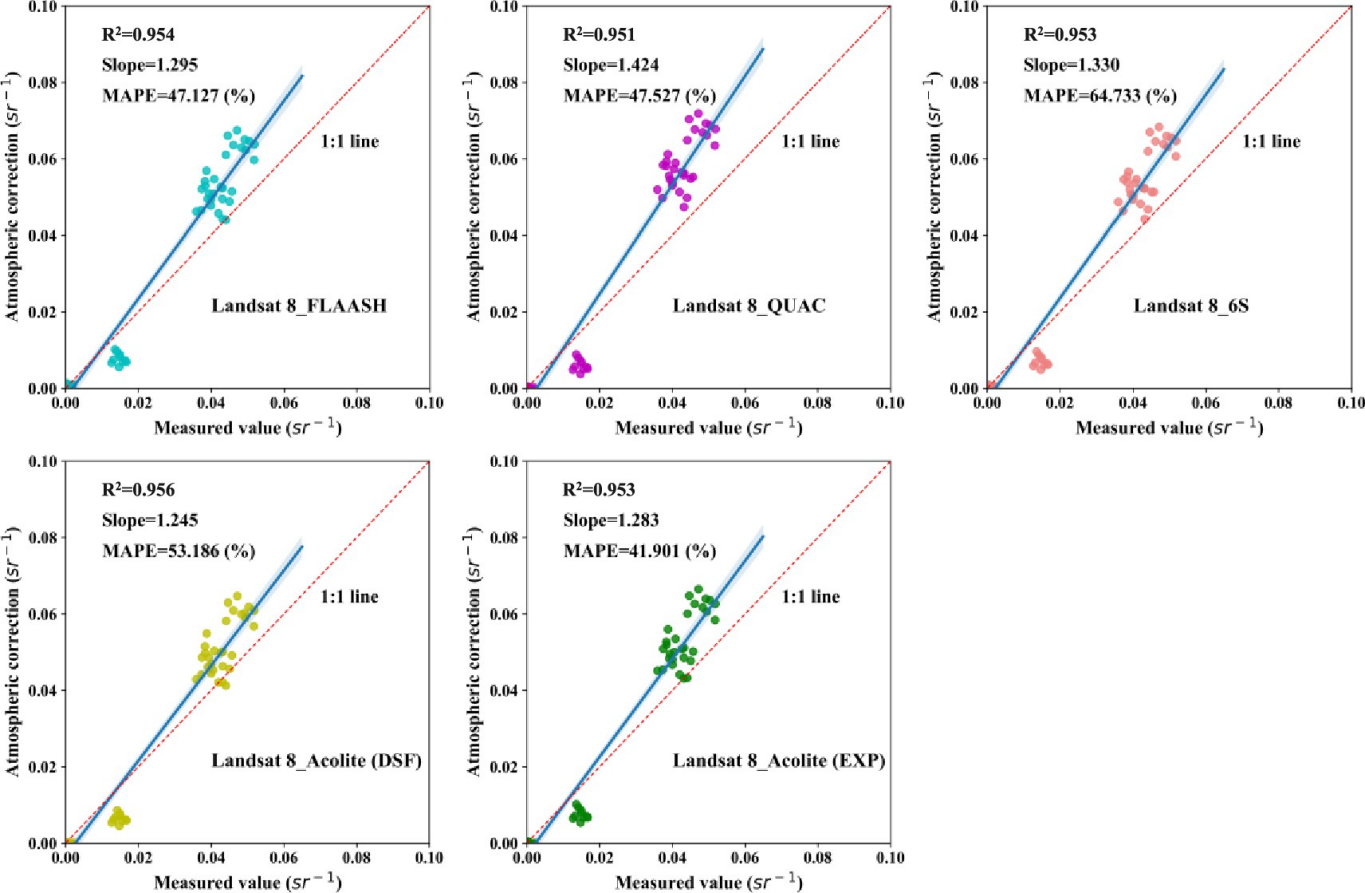
Landsat 8 atmospheric correction accuracy scatter plot.

**Fig 4:**
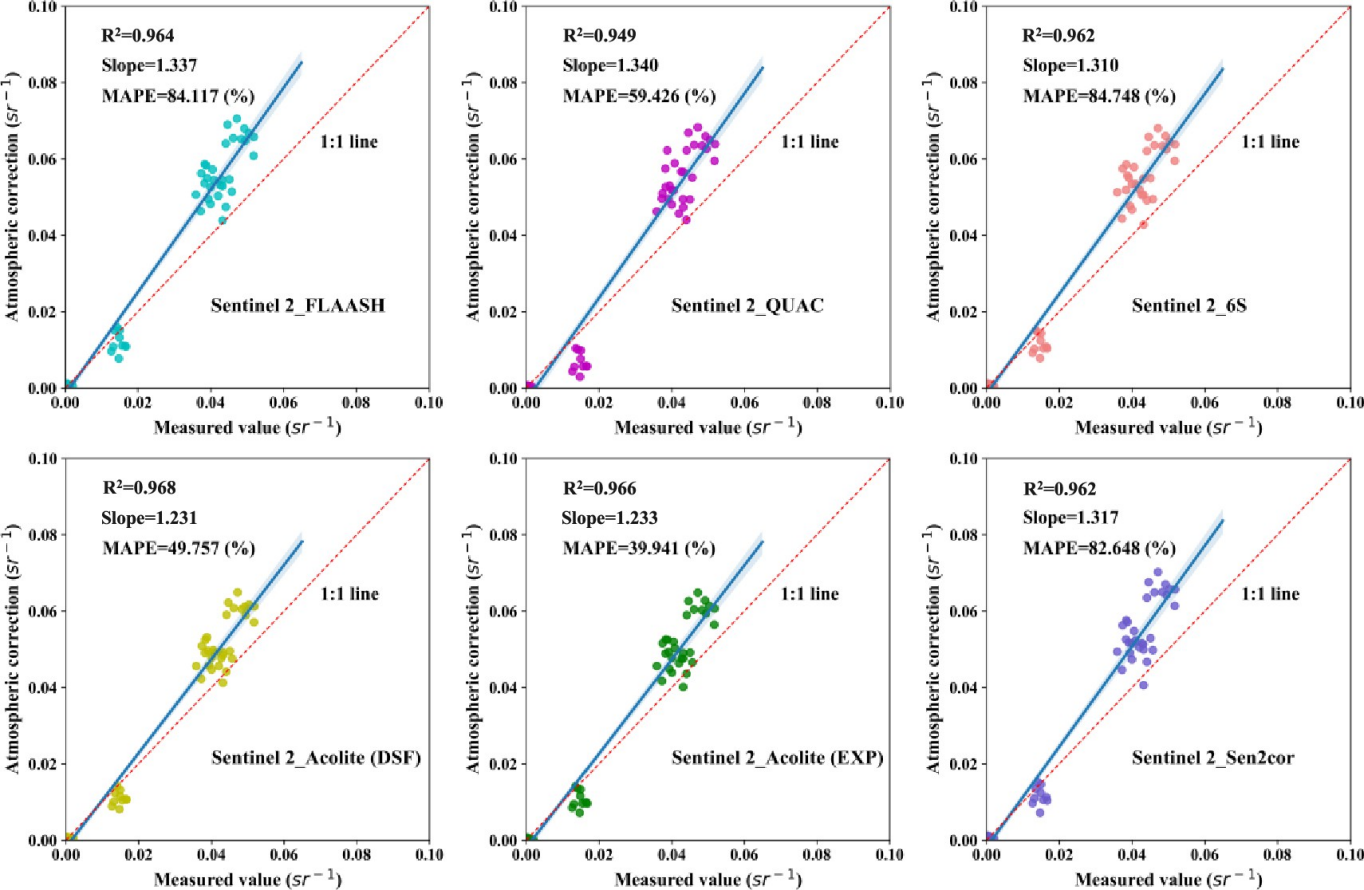
Sentinel 2 atmospheric correction accuracy scatter plot.

To further quantify the overall accuracy of each atmospheric correction algorithm, this study selected ten sampling points of measured hyperspectra. Then, it drew the scatter plots of image reflectance and measured hyperspectral reflectance at the center wavelengths of the six bands. The *R*^*2*^, *Slope*, and *MAPE* indicators were used to evaluate the correction effect quantitatively. Figs 3 and 4 show the scatterplot of atmospherically corrected reflectance versus measured values for Landsat 8 and Sentinel 2 images, respectively.

Fig 3 shows the correlation between the atmospherically corrected reflectance values based on Landsat 8. The measured hyperspectral reflectance values are all high, with *R*^*2*^ above 0.951. The *Slope* of the fitted lines is all greater than 1, with a 1.245−1.424 range. The results indicate that the measured values are greater than the atmospherically corrected reflectance values in the low reflectance region. In comparison, the measured values are less than the atmospherically corrected reflectance values in the high reflectance region, consistent with the findings shown in Fig 2. The *MAPE* values, distributed between 41.901−64.733%, vary more drastically compared to *R*^*2*^ and *Slope*. Although the *MAPE* of Acolite-DSF is not the smallest value, it has the largest *R*^*2*^ and the smallest *Slope*. Combining the three evaluation indexes, the Acolite-DSF atmospheric correction algorithm demonstrates the best correction effect.

Fig 4 shows the correction effect of the atmospheric correction algorithms based on Sentinel 2. The *R*^*2*^ of the corrected reflectance values to the measured values of each algorithm are larger and distributed between 0.949−0.968. The *Slope* is 1.231−1.337, while *MAPE* is distributed between 39.941−84.748%. In comparison, Acolite-DSF shows the best correction, consistent with the findings in Fig 2. In addition, this study extracted the reflectance values of Sentinel 2 reflectance product data (atmospheric correction algorithm is Sen2cor) at the center wavelengths of the six bands using the 10 sampling points mentioned above. The evaluation metrics were inferior to Acolite-DSF in all cases. The results indicate that the Acolite-DSF atmospheric correction algorithm is more suitable for atmospheric correction of the Ebinur Lake water body.

Combining Figs 3 and 4 shows that the Acolite-DSF atmospheric correction algorithm is better than other algorithms for both Landsat 8 and Sentinel 2 for the waters of Ebinur Lake, i.e., Acolite-DSF is the suitable atmospheric correction algorithm for the study area.

### 3.2 Consistency of atmospheric correction algorithms

**Fig 5:**
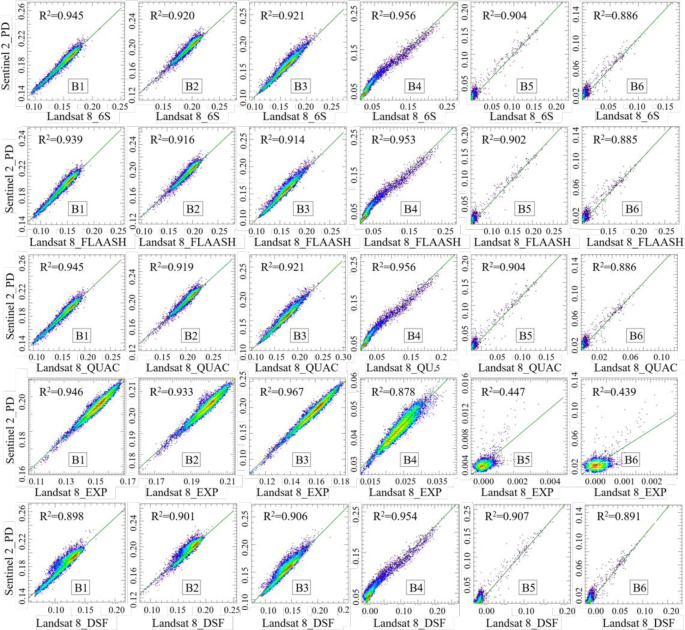
Scatter plot of Landsat 8 atmospheric correction imagery and Sentinel 2 reflectance products.

**Fig 6:**
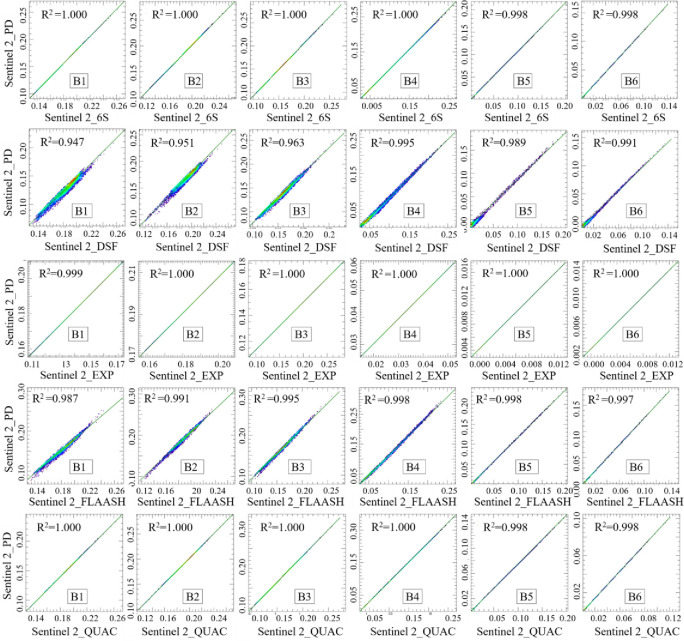
Scatter plot of Sentinel 2 atmospheric correction imagery and Sentinel 2 reflectance products.

The time-series nature of remotely sensed data is one of the main reasons it can be widely used in water-environmental monitoring. However, realizing remote sensing monitoring on time series inevitably involves different sensor types. Therefore, the consistency of atmospheric correction applied to different sensors is extremely important for accurately monitoring water-environmental parameters. To explore the consistency of the atmospheric correction algorithms applied to different sensors, this study resampled to 30 m the Sentinel 2 reflectance product data on May 19, 2021. Its pixel-by-pixel scatter plots against the atmospherically corrected image were plotted using the 2D Scatter Plot in the ENVI toolbox (Figs 5 and 6). Moreover, *R*^*2*^ was used to quantitatively reflect the consistency of the atmospherically corrected image with the reflectance products. By comparing the difference in *R*^*2*^ between the Landsat 8 and Sentinel 2 atmospherically corrected images and the product data, the atmospheric correction method applicable to Ebinur Lake and different sensors was finalized.

Fig 5 shows a scatter plot of the Landsat 8 atmospherically corrected image against the Sentinel 2 reflectivity product. Overall, *R*^*2*^ in the B5 and B6 bands is smaller than the first four bands. The Landsat 8_EXP and 8_DSF atmospheric corrections give slightly worse results than other corrections. The *R*^*2*^ is above 0.878 for all but the B5 and B6 bands of Landsat 8_EXP. Reflectivity anomalies in shallow water may cause a weak correlation for the B5 and B6 bands of Landsat 8_EXP after masking off the land in the connecting area of the large and small lakes.

Fig 6 shows that *R*^*2*^ between the atmospherically corrected Sentinel 2 and the Sentinel 2 reflectivity products is better than the atmospherically corrected *R*^*2*^ of the Landsat 8. This may be because the satellite parameters of the Landsat 8 satellite are different from the Sentinel 2, or there may be a difference in the observation geometry of the two sensors. The Sentinel 2_6S, Sentinel 2_EXP and Sentinel 2_QUAC corrections are very close to the Sentinel 2 reflectivity product data. Overall, *R*^*2*^ in the B5 and B6 bands is smaller than the first four bands. Unusually high agreement between Sentinel 2_EXP and the product data may be related to the atmospheric correction (Sen2cor) method for the Sentinel 2 reflectance product.

In order to quantitatively agree on each atmospheric correction algorithm applied to different sensors, this study calculated the difference of the scatterplot R^2^ in the corresponding bands based on the scatterplots of Sentinel 2, Landsat 8 with atmospherically corrected images, and Sentinel 2 reflectance product (Table 1).

**Table 1:** Difference in R^2^ between Sentinel 2, Landsat 8 and reflectance products.

Atmospheric correction algorithm	B1	B2	B3	B4	B5	B6
6S	0.055	0.080	0.079	0.044	0.094	0.112
QUAC	0.055	0.081	0.079	0.044	0.094	0.111
FLAASH	**0.048**	0.075	0.081	0.045	0.096	0.112
Acolite-DSF	0.049	**0.050**	0.057	**0.041**	**0.082**	**0.100**
Acolite-EXP	0.054	0.067	**0.033**	0.122	0.553	0.561

Table 1 indicates that among the six bands, only the R^2^ difference between the FLAASH atmospherically corrected B1 and the Acolite-EXP atmospherically corrected B3 is smaller than that of Acolite-DSF. The R^2^ differences of the remaining four bands are larger than Acolite-DSF. This indicates that the Acolite-DSF atmospheric correction algorithm is more consistent across sensors.

### 3.3 Atmospheric correction effect for each band

**Fig 7:**
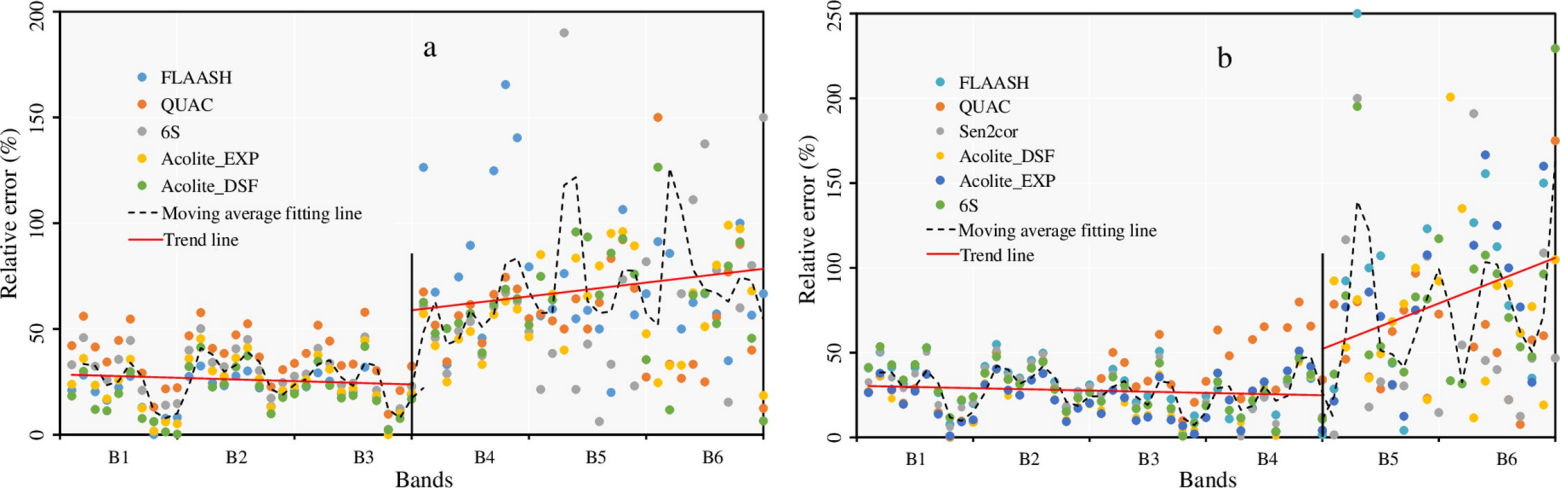
Relative error distribution (a and b indicate the relative error of Landsat 8 and Sentinel 2 atmospherically corrected reflectance to the measured hyperspectra, respectively).

To quantitatively characterize the atmospheric correction effect of each band, this study calculated the relative error between their atmospherically corrected reflectance values and measured hyperspectral values. Thus, the atmospheric correction effect of each band could be demonstrated. The distribution of the relative error of atmospheric correction for Landsat 8 and Sentinel 2 for each band is shown in Fig 7 (The original data comes from S1 Appendix).

Fig 7 indicates that the relative errors between the reflectance values based on Landsat 8 atmospheric correction and the measured values in each band are smaller and tend to decrease in the first three bands, with an average value of about 0.3. The relative errors in the last three bands are larger than the first three bands and tend to increase, with an average value of about 0.7, while the relative error in the B4 band is about 0.5. Compared with the first three bands, the relative errors of the last three bands fluctuate more drastically. For the relative errors of Sentinel 2 bands after atmospheric correction, the values of the first four bands are smaller and still show a decreasing trend. Their average values are also around 0.3. The relative errors of the last two bands fluctuate sharply, and the upward trend is more obvious. This may be caused by the significant difference between the strong absorption of the water body and the high reflection of the land at the shortwave infrared band in the land-water interface area.

## 4. Discussion

### 4.1 Suitability of atmospheric correction algorithms to Ebinur Lake’s water

**Fig 8:**
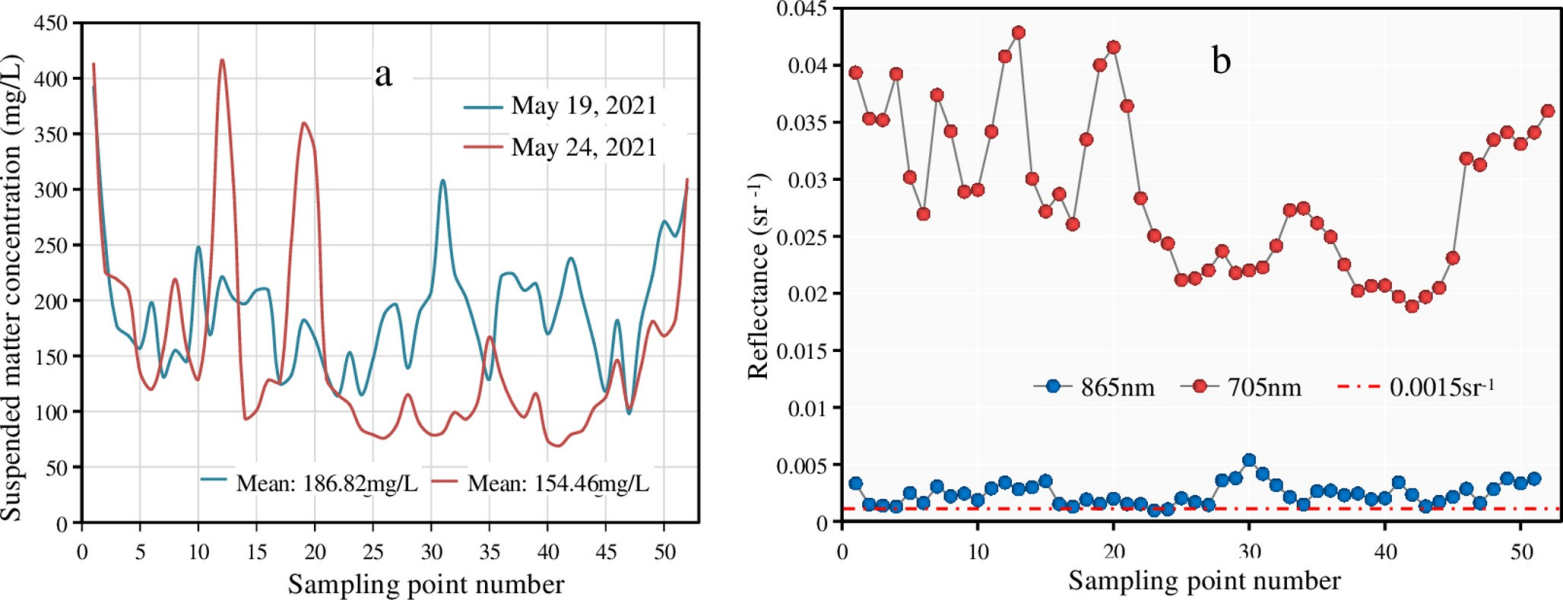
Characterization of Ebinur Lake waters (a: Suspended particulate matter concentration; b: NIR band reflectance level).

According to two successive field surveys, the mean concentration of suspended particulate matter (SPM) in the Ebinur Lake water body was 186.82 mg/L and 154.46 mg/L, respectively (Fig 8A). The results indicate the very turbid water, corroborated by its transparency of 17.51 cm. In addition, this study examined the reflectance of Landsat 8 and Sentinel 2 images separately in the NIR band (Fig 8B). Fig 8B shows that the volume sensor reflectance values of the NIR band are almost always above 0.0015 *sr* ^-1^. This value reflects the high turbidity of Ebinur Lake’s water, bringing a strong reflectance [17]. This prompted us to explore the effectiveness of land-oriented atmospheric correction methods applied to Ebinur Lake’s water column. Therefore, this study selected various atmospheric correction methods for water bodies and land to conduct atmospheric corrections for Ebinur Lake’s water body.

Ebinur Lake is adjacent to the Alashankou area, beset with exceptionally strong and prevalent wind. The waves caused by wind action often form flares (Glint) on the image. Acolite-DSF uses the darkest pixels in the field of view as a reference and can effectively attenuate the effects of solar flares [46, 47]. In addition, Acolite-DSF sets three aerosol types (marine, terrestrial, and urban): aerosol optical thickness, atmospheric pressure, and multiple imaging geometries. Choosing appropriate parameters makes its atmospheric correction results for Ebinur Lake’s water more satisfactory. In contrast, 6S ignores directional and near-neighbor effects [45]. FLAASH and QUAC assume no near-neighbor effect of image elements [45]. Acolite-EXP’s required assumption of zero reflectivity in the NIR band does not align with the characteristics of Ebinur Lake’s water [51]. Acolitie_DSF may have limitations in areas with little variation in water depth [52]. According to field data, the depth of Ebinur Lake varies greatly (with many reefs and small islands), which may be the main reason for the better atmospheric correction effect of Acolitie_DSF in the Ebinur Lake. Therefore, for shallow, turbid saltwater lakes in arid areas, the use of Acolite_DSF may be able to obtain more accurate water surface reflectance. This is consistent with previous research [14, 53].

### 4.2 Suitability of atmospheric correction algorithms for time-series water color inversion in Ebinur Lake

Atmospheric correction is the key basis for remote sensing of water color in lakes. The time series monitoring of water color parameters facilitates the realization of the environmental recovery of rivers and lakes. However, such monitoring inevitably requires the joint use of different sensors. Findings identified Acolite-DSF as the most suitable atmospheric correction algorithm for Ebinur Lake’s water body. However, the consistency of atmospheric correction for different sensors is still unclear. The Landsat series of sensors have similar physical parameters and high reflectance consistency [36]. Therefore, this study examined the consistency of reflectance after atmospheric correction by Acolite-DSF using the OLI sensor of Landsat 8 and the MSI sensor of Sentinel 2. Landsat 8 images with time October 15, 2017, and Sentinel 2 images with time October 14, 2017, were acquired for Ebinur Lake with a 1-day transit interval. However, the water conditions of Ebinur Lake may vary dramatically within one day.

**Fig 9:**
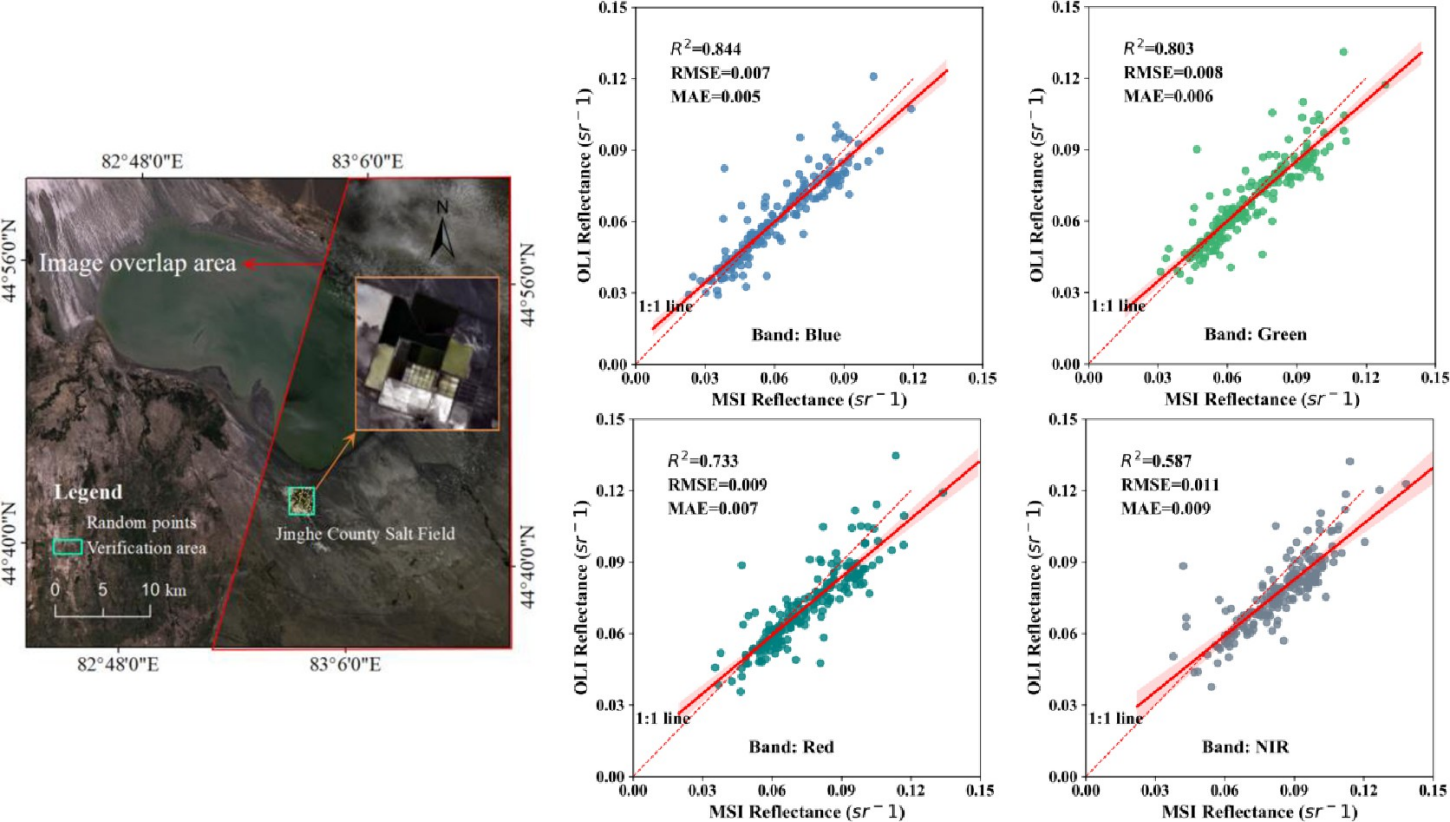
Consistency test of reflectance in different images after Acolite-DSF atmospheric correction (The acquisition address of the remote sensing image come from http://viewer.nationalmap.gov/viewer/ and https://www.usgs.gov/centers/eros).

To overcome the above possible source of error, this study chose a salt farm area on the lake’s south shore with relatively stable water conditions for validation (Fig 9). This chosen water body, similar in water conditions to the Ebinur Lake, accounts for more than 50% of the total water area. The reflectance scatter plots of the blue-green-red and NIR bands of the two sensors after atmospheric correction are shown in Fig 9B–9E. The reflectance consistency of the first three bands of the Acolite-DSF atmospherically corrected volume sensor is high (*R*^*2*^ ≥ 0.733). In contrast, the *R*^*2*^ in the NIR band is only 0.587, which is less consistent. The results of this study are consistent with the atmospheric correction results of other turbid water bodies [54, 55]. Therefore, the Acolite-DSF atmospheric correction algorithm is deemed suitable for the time-series remote sensing monitoring of water color parameters in Ebinur Lake. However, the first three bands should be selected as far as possible to construct the inversion model.

### 4.3 Uncertainty analysis to identify atmospheric correction algorithm

Atmospheric correction is crucial for remote sensing monitoring of the water environment in inland lakes [36]. Amongst many atmospheric correction algorithms [56], the five selected in this study are characterized by ease of operation. They can be quickly implemented for atmospheric correction of water bodies. The suitability of other algorithms for Ebinur Lake’s water has not been explored. Therefore, there is uncertainty in identifying the optimal atmospheric correction algorithm for Ebinur Lake. In future studies, it may be possible to compare the efficacy and accuracy of more algorithms. Moreover, exploring their appropriateness may identify algorithms for specific types of lake waters.

At the reference data level, this study used the measured hyperspectral data as a reference and found that Acolite-DSF is more suitable for Ebinur Lake. However, the relatively small number of field-measured hyperspectral points (10 points) may bring uncertainty to validating the atmospheric correction accuracy. Future studies can increase the number of field-measured hyperspectral locations and distributed uniformly in the evaluated waters.

In analyzing the consistency of algorithms on different remote sensing data, this study used the Sentinel 2 reflectance product as a reference and concluded that Acolite-DSF applied to Landsat 8 and Sentinel 2 had better consistency. However, the Sentinel 2 reflectance product was produced using the Sen2cor atmospheric correction algorithm, which has some limitations [57, 58]. Therefore, some uncertainties remain in the consistency test. More reliable conclusions may be obtained in future studies using measured hyperspectral data as a reference.

In addition, the two types of remote sensing data selected in this study have different transit times at Ebinur Lake and theoretically have different atmospheric conditions. The small time difference between the transit of the two remotely sensed images and the similarity of the atmospheric conditions may introduce uncertainty in the optimization of the atmospheric correction algorithm. More remote sensing data can be introduced in future studies to obtain more convincing atmospheric correction algorithms for Ebinur Lake.

## 5. Conclusions

This study applied atmospheric corrections to the Landsat 8 and Sentinel 2 L1 level images of Ebinur Lake on May 19, 2021, using FLAASH, QUAC, 6S, Acolite (DSF, EXP), and Sen2cor, respectively. Sentinel 2 reflectance product data were used to verify the consistency of each atmospheric correction algorithm applied to the above two sensors. Combined with the quasi-synchronized measured hyperspectral data, the atmospheric correction methods applicable to the waters of Ebinur Lake were explored. The following conclusions were obtained:

1. Compared with the measured hyperspectral data, the atmospheric correction accuracy of Acolite-DSF is superior to FLAASH, QUAC, 6S, and Acolite-EXP. The Acolite atmospheric correction algorithm is more suitable for atmospheric correction of Ebinur Lake.
2. The bands with smaller errors after atmospheric correction in Landsat 8 are Blue, Green, and Red (with a relative error of about 0.3), and the relative error in the NIR band is about 0.5. The bands with smaller errors after atmospheric correction in Sentinel 2 are Blue, Green, Red, and NIR (around 0.3). In subsequent research, the Blue, Green, Red, and NIR bands of Landsat 8 and Sentinel 2 data can be used to conduct temporal monitoring of water-environmental parameters in Ebinur Lake.
3. Acolite-DSF has good consistency in the atmospheric correction of images acquired by different Landsat 8 and Sentinel 2 sensors compared to other atmospheric correction algorithms. This algorithm can be used for atmospheric correction of different sensors.

This study provided a practical reference for atmospheric correction in remote sensing monitoring water-environmental parameters in salt lakes. The findings have practical significance for restoring the regional lake water environment.

## Supporting information

S1 Appendix (DOCX)
