# Supplementary material for: Evaluation of atmospheric correction algorithms for salt lake water assessment: Accuracy, band-specific effects, and sensor consistency
Source: PLoS One. 2024 Dec 23;19(12):e0315837. doi: 10.1371/journal.pone.0315837 (PMC11666065; doi:10.1371/journal.pone.0315837)
Supplement: S1 Appendix — (DOCX) [file pone.0315837.s001.docx]

**Evaluation of Atmospheric Correction Algorithms for Salt Lake Water Assessment: Accuracy, Band-Specific Effects, and Sensor Consistency**

Changjiang Liu ^a,b^, Fei Zhang ^c*^, Chi-Yung Jim ^d^, Saheed Adeyinka Oke ^e^, Elhadi Adam ^f^

^a^ Xinjiang Laboratory of Lake Environment and Resources in Arid Zone, Urumqi 830054,China

^b^ College of Geographic Science and Tourism, Xinjiang Normal University, Urumqi 830054, China

^c^ College of Geography and Environmental Sciences, Zhejiang Normal University, Jinhua 321004, China

^d^ Department of Social Sciences, Education University of Hong Kong, Lo Ping Road, Tai Po, Hong Kong, 999077, China

^e^ Civil Engineering Department, Central University of Technology, Bloemfontein, 9300, South Africa

^f^ School of Geography, Archaeology and Environmental Studies, University of the Witwatersrand, Johannesburg, 2050, South Africa

^*^ Corresponding author: zhangfei3s@163.com or [zhangfei3s@zjnu.edu.cn](mailto:zhangfei3s@zjnu.edu.cn)

**S1 Appendix**

This section contains “The minimal data set” that are used throughout this paper. The minimal data set in the below table is the original data in Figure 7 of the manuscript.

| Relative error | | | | | | |
| --- | --- | --- | --- | --- | --- | --- |
| Bands | FLAASH | QUAC | 6S | Acolite_EXP | Acolite_DSF | Mean |
| b1 | 0.211 | 0.421 | 0.331 | 0.239 | 0.183 | 0.277 |
| b1 | 0.282 | 0.560 | 0.459 | 0.361 | 0.300 | 0.392 |
| b1 | 0.203 | 0.415 | 0.324 | 0.235 | 0.120 | 0.259 |
| b1 | 0.163 | 0.344 | 0.260 | 0.170 | 0.113 | 0.210 |
| b1 | 0.223 | 0.446 | 0.356 | 0.259 | 0.195 | 0.296 |
| b1 | 0.276 | 0.547 | 0.446 | 0.357 | 0.298 | 0.385 |
| b1 | 0.130 | 0.292 | 0.210 | 0.126 | 0.077 | 0.167 |
| b1 | 0.001 | 0.131 | 0.062 | 0.017 | 0.062 | 0.055 |
| b1 | 0.078 | 0.217 | 0.140 | 0.061 | 0.014 | 0.102 |
| b1 | 0.082 | 0.222 | 0.147 | 0.052 | 0.003 | 0.101 |
| b2 | 0.276 | 0.468 | 0.400 | 0.360 | 0.322 | 0.365 |
| b2 | 0.325 | 0.577 | 0.502 | 0.454 | 0.413 | 0.454 |
| b2 | 0.244 | 0.408 | 0.342 | 0.302 | 0.226 | 0.304 |
| b2 | 0.232 | 0.385 | 0.321 | 0.277 | 0.240 | 0.291 |
| b2 | 0.278 | 0.472 | 0.406 | 0.363 | 0.321 | 0.368 |
| b2 | 0.301 | 0.525 | 0.450 | 0.411 | 0.373 | 0.412 |
| b2 | 0.221 | 0.367 | 0.304 | 0.264 | 0.230 | 0.277 |
| b2 | 0.134 | 0.227 | 0.173 | 0.130 | 0.099 | 0.153 |
| b2 | 0.187 | 0.308 | 0.247 | 0.209 | 0.176 | 0.225 |
| b2 | 0.207 | 0.337 | 0.277 | 0.227 | 0.193 | 0.248 |
| b3 | 0.227 | 0.385 | 0.287 | 0.260 | 0.233 | 0.278 |
| b3 | 0.293 | 0.518 | 0.408 | 0.376 | 0.347 | 0.388 |
| b3 | 0.254 | 0.443 | 0.338 | 0.310 | 0.233 | 0.316 |
| b3 | 0.193 | 0.327 | 0.235 | 0.203 | 0.174 | 0.226 |
| b3 | 0.199 | 0.334 | 0.243 | 0.219 | 0.186 | 0.236 |
| b3 | 0.319 | 0.579 | 0.463 | 0.445 | 0.418 | 0.445 |
| b3 | 0.176 | 0.303 | 0.210 | 0.185 | 0.161 | 0.207 |
| b3 | 0.024 | 0.097 | 0.025 | 0.000 | 0.023 | 0.034 |
| b3 | 0.113 | 0.209 | 0.123 | 0.100 | 0.077 | 0.125 |
| b3 | 0.190 | 0.324 | 0.229 | 0.194 | 0.169 | 0.221 |
| b4 | 1.264 | 0.675 | 0.609 | 0.573 | 0.627 | 0.749 |
| b4 | 0.674 | 0.518 | 0.458 | 0.421 | 0.479 | 0.510 |
| b4 | 0.331 | 0.345 | 0.289 | 0.250 | 0.502 | 0.343 |
| b4 | 0.746 | 0.563 | 0.490 | 0.452 | 0.528 | 0.556 |
| b4 | 0.895 | 0.616 | 0.535 | 0.488 | 0.574 | 0.622 |
| b4 | 0.457 | 0.433 | 0.375 | 0.332 | 0.388 | 0.397 |
| b4 | 1.248 | 0.663 | 0.605 | 0.569 | 0.618 | 0.741 |
| b4 | 1.655 | 0.745 | 0.671 | 0.632 | 0.688 | 0.878 |
| b4 | 1.404 | 0.689 | 0.630 | 0.594 | 0.644 | 0.792 |
| b4 | 0.794 | 0.549 | 0.487 | 0.463 | 0.519 | 0.562 |
| b5 | 0.563 | 0.571 | 0.214 | 0.852 | 0.749 | 0.590 |
| b5 | 0.594 | 0.538 | 0.385 | 0.664 | 0.637 | 0.564 |
| b5 | 0.762 | 0.500 | 1.900 | 0.400 |  | 1.795 |
| b5 | 0.548 | 0.643 | 0.214 | 0.835 | 0.959 | 0.640 |
| b5 | 0.588 | 0.500 | 0.429 | 0.655 | 0.935 | 0.621 |
| b5 | 0.500 | 0.625 | 0.062 | 0.798 | 0.661 | 0.529 |
| b5 | 0.200 | 0.833 | 0.333 | 0.952 | 0.859 | 0.635 |
| b5 | 1.065 | 0.922 | 0.734 | 0.960 | 0.927 | 0.922 |
| b5 | 0.567 | 0.692 | 0.231 | 0.893 | 0.759 | 0.628 |
| b5 | 0.667 | 0.273 | 0.818 | 0.477 | 0.355 | 0.518 |
| b6 | 0.913 | 1.500 |  | 0.246 | 1.265 |  |
| b6 | 0.857 | 0.333 |  | 0.327 | 0.118 | 1.260 |
| b6 | 0.500 | 0.267 | 0.667 | 0.329 |  | 0.882 |
| b6 | 0.625 | 0.333 | 1.111 | 0.671 | 0.660 | 0.680 |
| b6 | 0.667 | 0.250 | 1.375 | 0.511 | 0.670 | 0.695 |
| b6 | 0.571 | 0.556 | 0.778 | 0.802 | 0.525 | 0.646 |
| b6 | 0.350 | 0.769 | 0.154 | 0.990 | 0.798 | 0.612 |
| b6 | 1.000 | 0.900 | 0.600 | 0.973 | 0.912 | 0.877 |
| b6 | 0.565 | 0.400 | 0.800 | 0.680 | 0.456 | 0.580 |
| b6 | 0.667 | 0.125 | 1.500 | 0.185 | 0.065 | 0.508 |
